# A Mobile Health App for Facilitating Disease Management in Children With Atopic Dermatitis: Feasibility and Impact Study

**DOI:** 10.2196/49278

**Published:** 2023-12-13

**Authors:** Alex Zvulunov, Stepan Lenevich, Natalia Migacheva

**Affiliations:** 1 Sheba Medical Center Reichman University, Herzliya Ramat Gan Israel; 2 AvantaTrading Ltd Moscow Russian Federation; 3 Department of Pediatrics Samara State Medical University Samara Russian Federation

**Keywords:** atopic dermatitis, eczema, Atopic App mobile health application, artificial intelligence, dermatitis, dermatology, skin, disease management, child, children, pediatric, pediatrics, feasibility, mHealth, mobile health, app, apps, applications, applications

## Abstract

**Background:**

Inadequate control of atopic dermatitis (AD) increases the frequency of exacerbations and reduces the quality of life. Mobile health apps provide information and communication technology and may increase treatment adherence and facilitate disease management at home. The mobile health app, Atopic App, designed for patients and their caregivers, and the associated web-based patient education program, Atopic School, provide an opportunity for improving patients’ and caregivers’ engagement and adherence to the management of AD.

**Objective:**

This noninterventional, observational study aimed to explore the feasibility and potential impact on the management of AD in children by caregivers using the Atopic App mobile health app.

**Methods:**

The patient-oriented eczema measure (POEM) and numerical rating scale for the grading of pruritus were used as severity scores (scale range: 0-28). The artificial intelligence model of the app was used to assess the severity of AD based on the eczema area and severity index approach. The deidentified data enabled the analysis of the severity of AD, treatment plan history, potential triggers of flare-ups, usage of available features of the app, and the impact of patient education.

**Results:**

During a 12-month period, of the 1223 users who installed the app, 910 (74.4%) registered users were caregivers of children with AD. The web-based Atopic School course was accessed by 266 (29.2%) caregivers of children with AD, 134 (50.4%) of whom completed the course. Usage of the app was significantly more frequent among those who completed the Atopic School program than among those who did not access or did not complete the course (*P*<.001). Users who completed a second POEM 21 to 27 days apart exhibited a significant improvement of AD severity based on the POEM score (*P*<.001), with an average improvement of 3.86 (SD 6.85) points. The artificial intelligence severity score and itching score were highly correlated with the POEM score (*r*=0.35 and *r*=0.52, respectively).

**Conclusions:**

The Atopic App provides valuable real-world data on the epidemiology, severity dynamics, treatment patterns, and exacerbation-trigger correlations in patients with AD. The significant reduction in the POEM score among users of the Atopic App indicates a potential impact of this tool on health care engagement by caregivers of children with AD.

## Introduction

Atopic dermatitis (AD) ranks highest among all skin disorders as a cause of lost disability-adjusted life-years in patients worldwide [1]. Poor medication adherence is a major barrier to treatment success in AD and results from various underlying causes, including forgetfulness, medication side effects, complex dosing regimens, cost barriers, a lack of understanding about the importance of the medication, and disruptions to daily activities [2,3]. Addressing these barriers often requires a multidisciplinary approach involving health care providers, parents, and the child, which is very time-consuming and imposes an additional barrier. A number of solutions have been offered to date to address barriers for poor medication adherence, such as patient education, medication simplification, and telemedicine. Research investigating action plans as educational tools for managing pediatric AD or teledermatology revealed that the effectiveness of these measures was either unexamined or considered insignificant [4,5], with a notable absence of reports on how treatment simplification impacts patients’ adherence. In a systematic review of 11 studies exploring the therapeutic adherence of mobile apps, 7 studies confirmed that use of the mobile app increased treatment adherence [6]. The use of mobile apps in AD is still uncommon, and few of these have been scientifically studied; existing studies have mainly demonstrated a given app’s feasibility and involved small numbers of users [7,8].

The use of artificial intelligence (AI) in mobile health apps for the management of AD involves machine learning algorithms and data analysis techniques to assist in various aspects of AD care. AI in these apps may help with tasks such as assessing the severity of AD, providing personalized treatment recommendations, predicting flare-ups, and enhancing the overall management and monitoring of the condition. AI-driven features aim to improve patients’ and caregivers’ involvement in the treatment process and to improve adherence.

The Atopic App was developed following a series of 20 in-depth interviews with dermatologists, allergists, adult patients with AD, and parents of children with AD that aimed to identify difficulties in the management of AD in home settings. The app uses a numerical rating scale for grading the severity of pruritus [9] and the patient-oriented eczema measure (POEM) [10] as a global severity score.

AI can assist in tailoring treatment plans for individuals with atopic eczema by analyzing patient data, including lifestyle factors, environmental conditions, and treatment responses. Attempts to develop accurate automated measurements of eczema severity using images have shown promising results. While impressive accuracy was achieved in the diagnosis and assessment of the severity of AD [11-13], to reap the technological benefits in remote patient monitoring and self-management, there is a need to validate the results of AI models on the images taken by patients in home settings.

Our app’s AI model was designed to evaluate the severity of AD using the eczema area and severity index (EASI) method. This AI severity estimation model was embedded into the Atopic App for the analysis of images taken by users at home.

The AI severity model was previously trained on publicly available digital images on the internet. An image search was conducted based on the keywords “eczema” or “atopic dermatitis” and reviewed by 2 certified dermatologists. Only images that were unequivocally considered representative of AD were used for the AI training. The affected area was marked by the dermatologists using the Computer Vision Annotation Tool (Intel) [14]. The images were split into training and testing sets. A HarDNet model [15] was trained to recognize skin versus nonskin, skin with signs of AD, and each sign along with the severity. The images were augmented using the *albumentations* library [16,17] and shrunk to 384 × 384 pixels prior to feeding to the model [17]. The intersection over union was used as a loss function [15].

The Atopic App [18] is available as a free download in the App Store and Google Play for patients with AD and their caregivers. A Chabot-guided onboarding process includes completion of the POEM questionnaire, acquisition of clinical photographs and the numerical rating scale for the severity of itch [19], transcription of action plans [20] prescribed by a treating physician, documentation of suspected triggers of exacerbations, and patient education by the web-based Atopic School program [21].

The purpose of this noninterventional, observational study was to assess the feasibility of the Atopic App mobile health app in terms of in-app retention and engagement, the accuracy of the AI algorithm, the impact on patients’ adherence, and subsequent disease severity in children with AD.

## Methods

### Ethical Considerations

The Institutional Helsinki Committee concluded that the research satisfied the criteria for exemption from additional review and a waiver for informed consent. All collected data were deidentified.

### Study Design

The study participants were users of the app who downloaded the free Atopic App between May 2021 and April 2022 and declared that they are parents or caregivers of a child with AD. The collected demographic data included the date of birth and gender of the person with AD.

The app reminded users to submit a subsequent POEM on the seventh day after completion of the previous POEM by daily push notifications until the form was submitted again.

AI severity scores were calculated for the images taken by users of the mobile app as follows: (1) the percentage of skin affected by AD on the image was calculated as a percentage of the number of pixels classified by the model as skin with signs of AD out of all pixels classified as skin on the image, (2) the percentage of the affected skin was converted into an area score from 0 to 6 based on the EASI scoring method, (3) the severity of each of the EASI categories was averaged for each image based on the number of pixels representing different severities, and (4) the final severity score was calculated for each image by multiplying the area by the severity score. The resulting severity scores ranged from 0 to 72.

### Statistical Analysis

The collected data were extracted using SQL queries from PostgreSQL tables and further refined with Python scripts [22]. The data were then analyzed using paired *t* tests, Mann-Whitney U tests, Wilcoxon signed-rank tests, Pearson correlations, and multiple regression.

For evaluation of patients’ adherence, the app users were divided into 3 groups. Cohort 1 included users who did not register for the Atopic School, cohort 2 included users who registered but did not complete the program, and cohort 3 included users who completed the program. Atopic School was considered completed if users scored ≥80% of correct answers in a quiz at the end of each of the following program sections: AD basics, skin care in AD, management of flares, and triggers of exacerbation.

AI accuracy in the determination of AD severity was determined using multiple regression analysis of POEM scores and AI-estimated severities from the submitted images. Only cases that had images obtained and submitted by users in the preceding 0 to 6 days were included in the analysis. Correspondingly, each POEM record was matched with an average AI severity and itch score recorded over the course of the 0 to 6 days preceding the POEM completion for a given user.

The following data were included in the multiple linear regression analysis of the relationship between POEM scores and independent variables: age, gender, recorded medications, recorded triggers, AI AD severity score, and itch score. An *F* test was used as a measure of a model’s accuracy on a data set.

To assess the impact of the app in a real-world context, POEM scores were categorized into 5 severity levels [23]: clear or almost clear (scores 0-2), mild (scores 3-7), moderate (scores 8-16), severe (scores 17-24), and very severe (scores 25-28).

## Results

**Figure 1:**
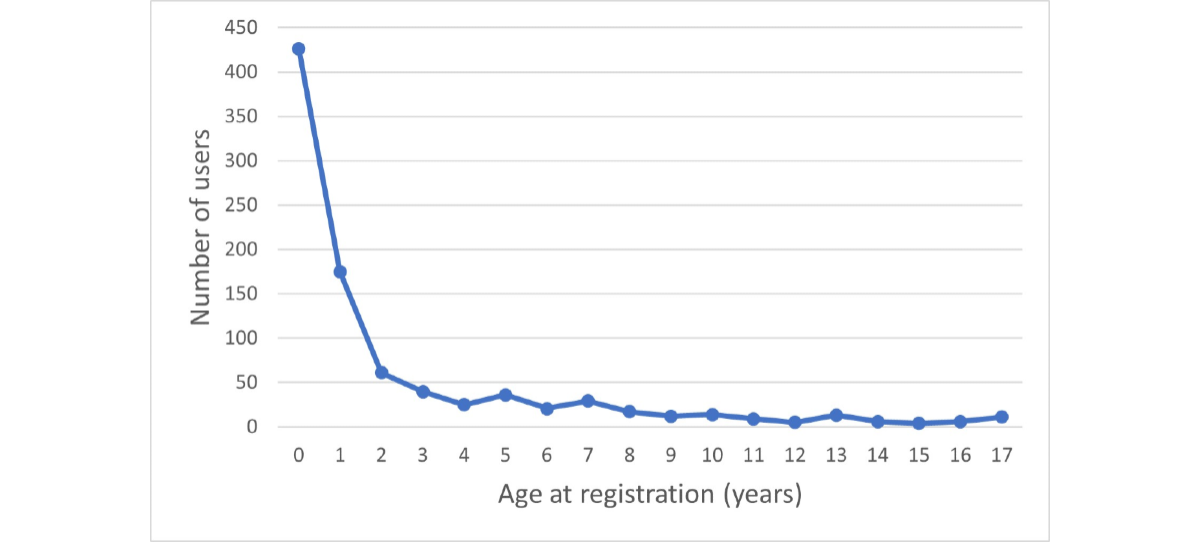
Age distribution of children with atopic dermatitis cared for by users of the Atopic App.

During the 12-month period from May 2021 to April 2022, 1223 people downloaded the app. Of these, 888 (72.6%) were parents of a child with AD, 224 (18.3%) were adults with AD, and 111 (9.1%) were other caregivers of patients with AD. In total, 910 (74.4%) registered users were caregivers of children with AD; of these children with AD, 494 (54.3%) were boys and 416 (45.7%) were girls. The age distribution of the registered children with AD is presented in Figure 1.

The period between the first and the last launch of the app ranged from 0 to 366 days, and 165 (18%) users used the app for more than 4 weeks. Of the 910 users who were caregivers of children with AD, the web-based Atopic School patient education course was accessed by 266 (29.2%) users, of which 132 (49.6%) did not complete the course (cohort 2) and 134 (50.4%) did complete the course (cohort 3). The remaining 644 (70.8%) users did not register for the education course (cohort 1). Users who completed the education course used the app twice as frequently during the 3 months after the installation of the app compared to those who did not take the education course (*P*<.001).

**Figure 2:**
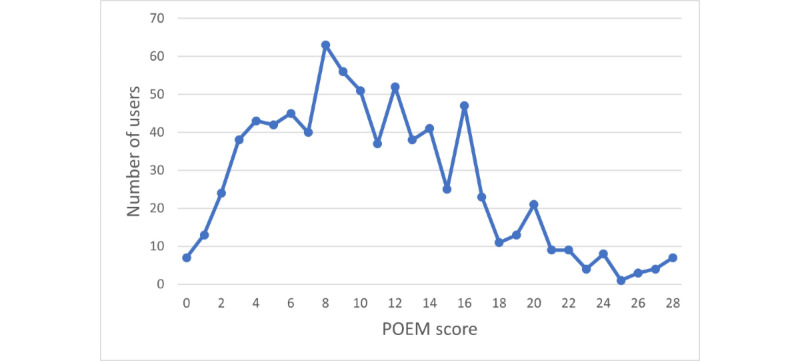
POEM score of children with atopic dermatitis cared for by users of the Atopic App at the time registration. POEM: patient-oriented eczema measure.

The POEM was completed at least once by 775 (85.2%) of the users who were caregivers of children with AD. The median POEM score at the time of registration in the application was 10 (IQR 6-14; range 0-28). The distribution of severity as measured by POEM score is presented in Figure 2.

**Figure 3:**
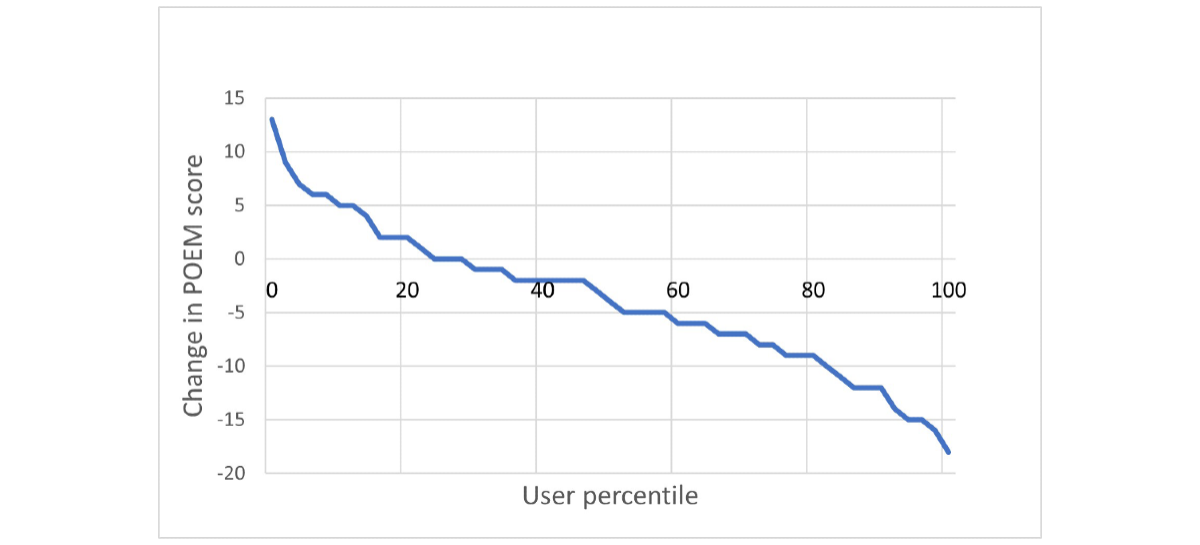
Change in POEM scores from day 1 to day 21-27 by user percentiles. POEM: patient-oriented eczema measure.

**Figure 4:**
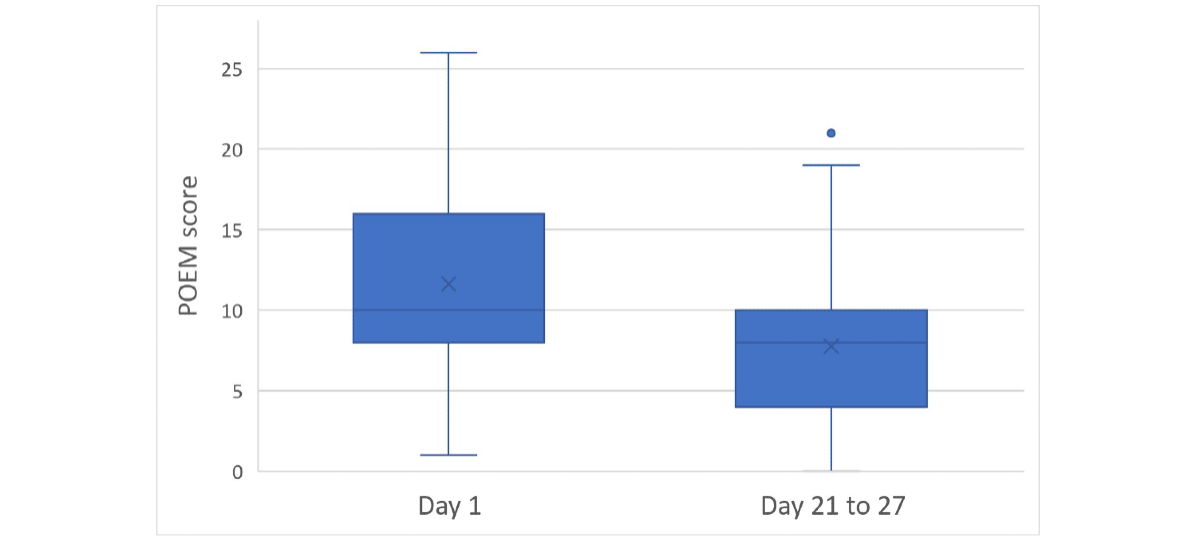
Distribution of POEM Scores at Day 1 and Days 21-27. The error bars represent the minimum and maximum values excluding outliers. POEM: patient-oriented eczema measure.

A total of 51 users completed a subsequent POEM 21 to 27 days apart from the first POEM. There was a statistically significant difference (*P*<.001) between the first and subsequent POEM scores. A decrease in the POEM score was observed for 36 (70.6%) users, while 3 (5.9%) users showed no difference and 12 (23.5%) users reported an increase in the subsequent POEM score (Figure 3). The average decrease in the POEM scores was 3.86 points *(P*=.001, Wilcoxon signed-rank test; Figure 4). A prominent shift from the more severe categories to the milder categories based on the POEM scores was observed from day 1 to days 21-27 (Table 1).

A total of 3385 photos were taken by 364 (40%) users. Of these, the data from 204 (56%) users were considered complete and were included in the multiple linear regression analysis to assess the relationship between POEM scores and several independent variables. Data from the remaining 160 (44%) users were excluded from the analysis because these users did not provide corresponding POEM scores after taking their photos. The AI severity and itching scores were highly significant predictors of POEM score (*P*<.001), while the presence of triggers (*P*=.14), medication records (*P*=.18), age (*P*=.29), and gender (*P*=.95) were weak predictors of POEM score, as expected. An *F* test was used as a measure of the model’s accuracy on the data set (multiple R=0.58, *F*_6,294_=25.2529, *P*<.001).

**Figure 5:**
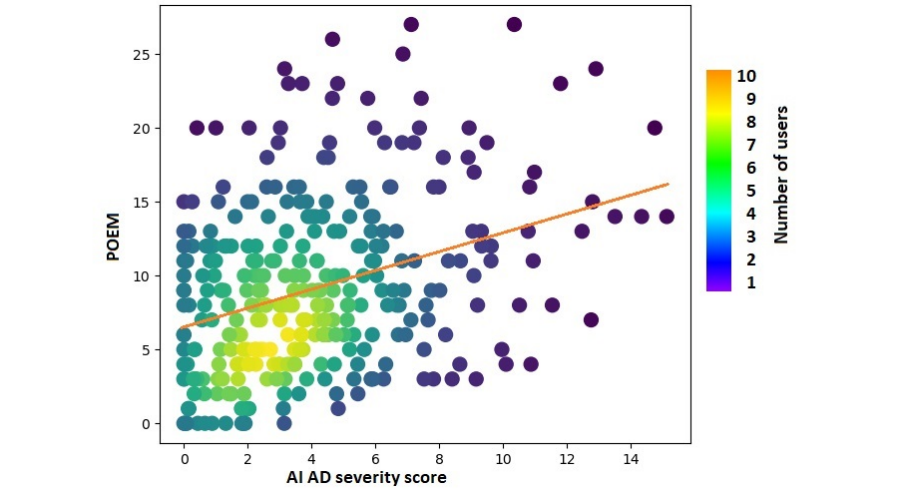
Normalized correlation between POEM and severity scores of AD predicted by AI. AD: atopic dermatitis; AI: artificial intelligence; POEM: patient-oriented eczema measure.

**Figure 6:**
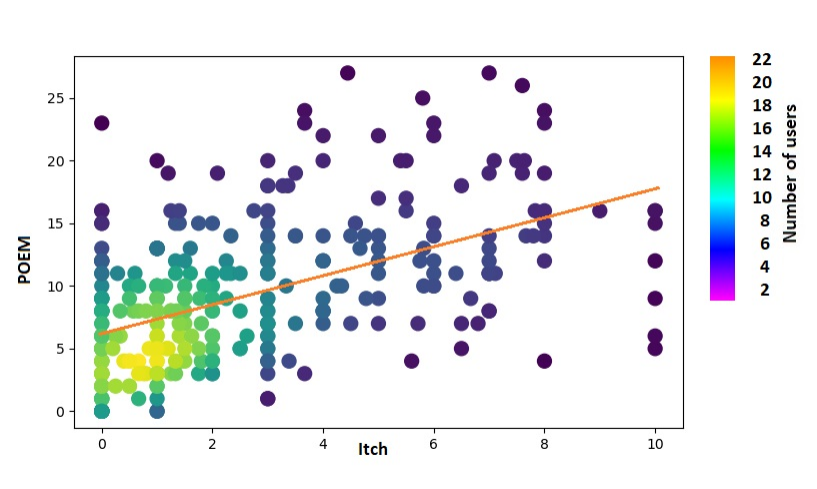
Normalized correlation between POEM and itch scores. POEM: patient-oriented eczema measure.

The AI severity score and itching score were highly correlated with the POEM score for photos taken during the 7 days leading up to and including the day of POEM completion (*r*=0.35 and *r*=0.52, respectively) (Figures 5 and 6). Conversely, age, gender, medications, and triggers were not significantly correlated with POEM scores (Table 2).

Among the 910 caregivers of children with AD, 330 (36.3%) documented prescribed action plans, which included using emollients (n=301, 91.2%), topical medication (n=126, 38.2%), and systemic medication (n=69, 20.9%). Topical corticosteroids were included in the action plans of 25.5% (n=84) of patients, while topical calcineurin inhibitors were included in 13.0% (n=43) of patients. Oral antihistamines were the most common systemic medication in children with AD (n=53, 16%). Systemic anti-inflammatory drugs were not prescribed in the action plans of any patient.

At least 1 potential trigger was suspected by 443 (48.7%) users (Table 3).

**Table 1:** A shift in severity categories based on patient-oriented eczema measure scores on day 1 and days 21 to 27.

Severity	Day 1, n	Days 21 to 27, n
Clear or almost clear	2	7
Mild	10	18
Moderate	31	22
Severe	7	4
Very severe	1	0

**Table 2:** Correlation coefficients between patient-oriented eczema measure scores and each independent variable.

Variable	*r*
Age	0.08
Gender	0.01
Recorded medications	0.11
Recorded triggers	–0.02
Average itching score	0.52
Average AI^a^ AD^b^ severity prediction score	0.35

^a^AI: artificial intelligence.

^b^AD: atopic dermatitis.

**Table 3:** Prevalent triggers suspected by users of the Atopic App.

Trigger	Users who added the trigger (n=443), n (%)
Dry air	275 (62.1)
Cow’s milk	195 (44)
Dust	191 (43.1)
Sweating	181 (40.9)
Pets	174 (39.3)
Heat	154 (34.8)
Stress	149 (33.6)
Detergents	141 (31.8)
Chicken eggs	138 (31.2)
Viral or bacterial diseases	102 (23)
Soap	80 (18.1)
Cereals	80 (18.1)
Synthetic clothing	74 (16.7)
Fish	73 (16.5)
Nuts	71 (16)
Cleaning products	58 (13.1)
Peanut	55 (12.4)
Seafood	47 (10.6)
Wool clothing	46 (10.4)
Tobacco smoke	46 (10.4)

## Discussion

### Principal Findings

There is a growing list of AD-related mobile apps [8]. Only a few of these are designed for bidirectional communication between patients or caregivers and the app, and they primarily focus on assessing the severity of the disease. The most important feature required for the development of mobile apps for caregivers of children with AD is an educational functionality, including knowledge of the disease, management of symptoms, medication usage, and triggers [8]. Unlike other mobile apps for AD management, the Atopic App contains Chabot-directed instruction of proper use of the app’s features and targeted education for enhancing adherence in the management of AD. The Atopic App also includes the basic features found in other apps. Other exceptional features of the app include its associated web-based patient-education program (Atopic School), AI assessment of severity, integration of action plans, and its ability to identify personal trigger factors for AD exacerbations.

Currently, there are no publications on the usability, acceptability, or impact of any of these apps in patients with AD or their caregivers, resulting in an inability to compare feasibility findings using our app to other available mobile health apps for AD. The most recent studies on mobile apps for AD focused on the burden of the disease as determined by itch score, quality of sleep, and energy or stress levels and involved a small number of users that precluded assessment of the clinical significance and impact of the app [7,24].

This study reports on the largest cohort of mobile app users in the management of AD. The similar correlations between POEM and itch scores and between POEM and AI severity scores indicate the usefulness and accuracy of the AI algorithm of the Atopic App in predicting AD severity.

The real-life impact demonstrated by the increase in the clear or almost clear POEM score category between day 1 and days 21 to 27 indicates that patients whose caregivers repeatedly used the app experienced a complete or nearly complete resolution of their symptoms, possibly related to improved engagement and adherence rates for prescribed treatments during the period of the study.

Environmental factors, like ambient climate conditions, dust, food allergens, pets, and clothing, can significantly impact AD flare-ups. The relevance of environmental factors can vary from person to person. Identifying and managing these triggers on an individual basis is essential for effectively controlling AD symptoms—this aspect of the management of AD was facilitated by the Atopic App.

The incorporation of novel technology has the potential to improve patients’ engagement and therapy adherence. It has been advised that easily implementable interventions should encourage parents and older children to take photographs and use clinician-designed apps that can deliver regular portable reminders with written plans of treatment regimens [20,25]. Documentation of prescribed action plans on the app may have improved treatment adherence, leading to an improvement in disease severity, at least among those users who had recorded sequential POEM scores in this study.

Nonadherence to therapy has been associated with poor therapeutic outcomes [26]. To improve adherence, health care providers are expected to motivate patients and their parents to adhere to treatment through appropriate patient-tailored education programs [27]. Clinical studies exploring the effects of mobile apps indicate that they promote treatment adherence [6].

Access to smartphones among adults is almost universal, and mobile app usage is also growing substantially, with adolescents representing the fastest growing sector to adopt smartphone technology [28].

### Limitations of the Study

There are multiple inherent limitations in studies that explore mobile health apps as auxiliary tools for optimizing outcomes in chronic diseases, including selection bias, a lack of control groups, unknown external confounding factors, and short observation periods.

Several user-driven factors affected the computation of the AI severity score, such as the distance between a smartphone camera and the affected skin, illumination conditions, and reporting bias, including selection of the area photographed. These factors, which potentially represent an obstacle for the reproducibility and comparability of AI severity estimations, should be addressed in the future by incorporating automated control over the illumination and distance during the process of photographing and providing clear instructions for the user.

The real-life impact demonstrated by the shift toward a milder severity of disease correlated with more frequent usage of the app may represent self-selection bias of a subset of highly motivated caregivers whose engagement and adherence levels may not be representative of the broader population of caregivers of children with AD. Additionally, there were possible obstacles for Atopic School attendance and completion that will be addressed in updated versions of the app. These include the lack of regular prompts to sign up for Atopic School and engage with the educational content and the dual registration process, whereby after initial registration within the app, logging in to the Atopic School course required a separate registration that demanded an additional effort and may have posed a barrier.

### Conclusion

The Atopic App is a valuable source of real-world data on the epidemiology, severity dynamics, treatment patterns, and exacerbation-trigger correlations in patients with AD. The Atopic App is a promising tool that can help increase the health care engagement of patients with AD and their caregivers. Addressing the limitations of this study in newer versions of the Atopic App can improve the feasibility and impact of the mobile health app for managing AD in children by caregivers.

## Abbreviations

AD: atopic dermatitis
AI: artificial intelligence
EASI: eczema area and severity index
POEM: patient-oriented eczema measure
